# Integrative phenotypic and functional genomic characterization of virulence and antimicrobial resistance in *Salmonella enterica* isolates from reptiles

**DOI:** 10.3389/fmicb.2026.1841627

**Published:** 2026-05-20

**Authors:** Dilan Amila Satharasinghe, Abraham Joseph Pellissery, Subhashinie Kariyawasam, Yugendar Reddy Bommineni, David Andrew Simon, Lijuan Zhou, Yevgeniya Abramzon, Danielle Stanek, Thomas Denagamage

**Affiliations:** 1 Large Animal Clinical Sciences, College of Veterinary Medicine, University of Florida, Gainesville, FL, United States; 2 Department of Basic Veterinary Sciences, Faculty of Veterinary Medicine and Animal Science, University of Peradeniya, Peradeniya, Sri Lanka; 3 Florida Department of Agriculture and Consumer Services, Bronson Animal Disease Diagnostic Laboratory, Kissimmee, FL, United States; 4 Department of Comparative, Diagnostic, and Population Medicine, College of Veterinary Medicine, University of Florida, Gainesville, FL, United States; 5 Florida Department of Health, Tallahassee, FL, United States

**Keywords:** antimicrobial resistance, public health, reptiles, *Salmonella*, virulence

## Abstract

The popularity of reptiles as exotic pets has increased over the years. Reptiles can harbor zoonotic pathogens, including *Salmonella*, posing a significant public health risk. This study evaluated the diversity of hosts affected by non-typhoidal *Salmonella* infections in reptiles, as well as the antimicrobial resistance (AMR), multidrug resistance (MDR), and virulence factor (VF) genes in whole-genome, plasmid DNA, and RNA in *Salmonella* isolated from reptiles in Florida, United States. Data on *Salmonella* culture testing from 2018 to 2025, available at the Bronson Animal Disease Diagnostic Laboratory, were analyzed for host diversity in *Salmonella* infections. Functional genomic analysis was conducted using whole-genome sequences (WGS), plasmid DNA, and RNA obtained from selected *Salmonella* isolates, targeting AMR and VF genes. The *Salmonella* culture case positivity rate in reptiles was 16.41% during the study period. The highest positivity percentage was observed in the order Squamata (35%), which includes lizards, dragons, iguanas, and snakes, followed by the orders Testudines and Crocodilia (12.2%). The antibiotic susceptibility testing of 24 *Salmonella enterica* isolates revealed that 58.3% were MDR and specifically resistant to beta-lactams (62.5%), aminoglycosides (62.5%), and tetracyclines (8.3%). Genomic analysis confirmed phenotypic AMR and revealed the presence of 55 AMR genes, with the majority showing resistance to fluoroquinolones (18.2%), carbapenems and quinolones (16.4%), tetracyclines and rifamycins (14.5%), amphenicols (12.7%), and other classes. The presence of the *tetA* gene in both the genomic and plasmid DNA of a tetracycline-resistant isolate highlighted reptiles’ role as stable zoonotic reservoirs for highly mobile genetic elements that can facilitate rapid horizontal gene transfer among pathogens. Transcriptomic analysis of isolates with MDR revealed differential expression patterns largely consistent with WGS analysis and identified additional AMR-related genes associated with MDR, efflux pumps, and membrane transport systems. A total of 239 VF genes were identified in isolates. Despite the health status of reptiles, the largest number of genes was associated with the Type III secretory system, invasion, motility, iron uptake, siderophore, fimbrial adherence, endotoxin, and lipopolysaccharides. Findings from this study underscore the importance of ongoing surveillance and improved hygiene practices when handling reptiles to reduce the risk of reptile-associated salmonellosis in humans.

## 1 Introduction

It has been reported that *Salmonella* causes 1.2 million illnesses and 450 deaths in the United States each year (Centers for Disease Control and Prevention, 2016). Although human salmonellosis is frequently associated with the ingestion of contaminated animal foods, contact with animals may also be a significant source of *Salmonella* infection (Musto et al., 2006). Hale et al. (2012) estimated that of the 445,213 human illnesses caused annually by seven groups of zoonotic pathogens, 127,155 were attributed to non-typhoidal *Salmonella* serovars associated with animal exposure. The popularity of reptiles as exotic pets has increased significantly over the years. However, these animals can harbor various zoonotic pathogens, including *Salmonella* species. The potential of reptiles to serve as reservoirs for *Salmonella* can pose a major public health risk, particularly to vulnerable populations, including children, older adults, and immunocompromised individuals. *Salmonella* has exhibited increasing resistance to commonly used antibiotics, causing challenges in both human and veterinary medicine. The emergence of antimicrobial resistance (AMR), including multidrug resistance (MDR), may further complicate treatment and control measures, necessitating comprehensive surveillance and mitigation strategies (Punchihewage-Don et al., 2024).

The identification of *Salmonella* virulence genes in both gastrointestinal (GIT) and systemic infection isolates recovered from reptiles highlights the potential zoonotic risks associated with reptile-associated *Salmonella*. Understanding the functions of these genes can provide valuable insights into how *Salmonella* strains adapt to different host environments and vary in disease severity (Kingsley and Bäumler, 2000).

Virulence genes present in *Salmonella* isolates from reptiles can be transmitted to human hosts through direct or indirect contact with these animals. The primary transmission route is fecal-oral, whereby *Salmonella*-contaminated reptile feces contaminate human hands, fomites, or food. Once introduced into the human GIT, these virulence genes enable *Salmonella* to adhere to intestinal epithelial cells, invade host tissues, and evade the immune response, potentially causing severe infections (Centers for Disease Control and Prevention, 2023). Therefore, the risk of transmission of virulent *Salmonella* or their virulence genes from reptiles to humans highlights the importance of proper hygiene practices when handling reptiles and their environments to reduce human salmonellosis (Centers for Disease Control and Prevention, 2023).

The impact of antimicrobial use on the emergence of AMR in both human and veterinary medicine is a growing concern (Bengtsson and Greko, 2014; Weese et al., 2015). AMR has been reported in a variety of free-ranging and captive reptiles (Cushing et al., 2011; Tang et al., 2020). Fluoroquinolones and third-generation cephalosporins are among the most commonly used antibiotics in reptile medicine (Isaza and Jacobson, 2013; Hernandez-Divers et al., 2017). However, practice guidelines for the first-line use of antibiotics for systemic infections in reptiles, pending antimicrobial susceptibility results, include potentiated sulfonamides, tetracycline, doxycycline, ampicillin, and aminoglycosides, namely gentamicin and amikacin (Hedley et al., 2021). The presence of AMR, including MDR in *Salmonella,* poses major challenges for both human and veterinary medicine. AMR reduces treatment efficacy, necessitating alternative or combined therapies, thereby increasing the treatment cost and complexity. Furthermore, some AMR genes are located on mobile genetic elements, such as plasmids and transposons, facilitating horizontal transfer of resistance determinants among bacteria (Partridge et al., 2018).

Many cases of reptile-associated salmonellosis go undiagnosed or unreported. For instance, a previous study estimated that exposure to reptiles and amphibians is associated with approximately 74,000 *Salmonella* infections annually in the United States, accounting for approximately 6% of all sporadic salmonellosis cases (Mermin et al., 2004). Similarly, a report by the Centers for Disease Control and Prevention (CDC) in the United States indicated that reptile-associated salmonellosis remains a considerable threat to human health, with approximately 93,000 (7%) cases of *Salmonella* infections each year attributable to contact with pet reptiles or amphibians (Bosch et al., 2016). Together, these reports suggest that the actual number of reptile-associated salmonellosis cases may be higher than reported, underscoring the potential underestimation of its true public health burden (Sodagari et al., 2020).

Therefore, the current study aimed to analyze AMR and virulence factor (VF) genes identified in *Salmonella* isolated from reptiles that died due to GIT or systemic infections, as well as from healthy reptiles, in Florida, United States, from 2018 to 2025.

## 2 Materials and methods

### 2.1 *Salmonella* culture and isolation

Non-typhoidal *Salmonella* strains (*n* = 33) isolated from captive reptile diagnostic submissions between 1 June 2018, and 30 June 2025, at the Bacteriology Division of the Bronson Animal Disease Diagnostic Laboratory (BADDL, Kissimmee, Florida) of the Florida Department of Agriculture and Consumer Services (FDACS) were used in the study. For *Salmonella* isolation, tissue or fecal samples were added to a tube with tetrathionate broth at ~1:10 ratio (e.g., 1 g of tissue/feces in 9 mL of tetrathionate broth) and incubated at 37 ± 2 °C for 24 h, followed by inoculation onto Xylose-Lysine-Tergitol 4 (XLT4) agar and Brilliant Green Agar (BGA) plates. Both agar plates were further incubated for 20–24 h at 37 ± 2 °C. After the incubation, presumptive *Salmonella* colonies were observed as entirely black, black with a yellow periphery, or pink-to-red with or without black centers on XLT4 agar plates, and pink-to-red colonies often surrounded by a red or reddish-pink halo on BGA plates. If the initial selective enrichment was negative for *Salmonella*, a delayed secondary enrichment (DSE) procedure was used in which the tetrathionate-enriched samples were kept at room temperature for 5–7 days followed by transferring 1 mL of the culture into a tube containing 10 mL of fresh tetrathionate enrichment broth, incubating at 37 °C ± 2 °C for 20 to 24 h and plating onto XLT4 and BG agar. The identity of the suspect *Salmonella* colonies was confirmed by the Bruker MALDI Biotyper Sirius System (Billerica, Massachusetts, United States). If the sample submitter requested, suspected colonies were serogrouped using the Wellcolex™ Color *Salmonella* Rapid Latex Agglutination test kit (ThermoFisher Scientific, Waltham, Massachusetts, USA) followed by serotyping at the reference laboratory (National Veterinary Services Laboratories, Ames, Iowa, USA). A total of 33 *Salmonella enterica* isolates were recovered from captive reptiles with GIT or systemic infections at BADDL during this period. Furthermore, eight fecal samples collected from apparently healthy reptiles, between 1 January 2025 and 30 June 2025, were also processed for *Salmonella* isolation. Two isolates, each from a captive reptile and a wild reptile that yielded *Salmonella,* were included in the study for comparison purposes.

### 2.2 Antibiotic susceptibility testing

During the study period, out of 33 reptile *Salmonella* isolates, 24 were subjected to antimicrobial susceptibility testing (AST) using the Sensititre^Tm^ Companion Animal Gram Negative COMPGN1F Vet AST Plate (ThermoFisher Scientific, Cleveland, Ohio, United States). The readings were interpreted according to the Clinical and Laboratory Standards Institute (CLSI) guidelines: CLSI M100TM Performance Standards for Antimicrobial Susceptibility Testing and CLSI VET01STM Performance Standards for Antimicrobial Disk and Dilution Susceptibility Tests for Bacteria Isolated from Animals M100Ed36E and VET01SEd7E (Clinical and Laboratory Standards Institute, 2024; Clinical and Laboratory Standards Institute, 2026). Isolates that showed resistance to three or more antimicrobial classes were considered as MDR isolates (Magiorakos et al., 2012).

### 2.3 While genome sequencing and data analysis

All *Salmonella* isolates (*n* = 33) collected during the study period were submitted for whole genome sequencing (WGS) through the Veterinary Laboratory Investigation and Response Network—*Salmonella* Sequence Project (Vet-LIRN-CVM-FDA)—coordinated by the Food and Drug Administration (FDA). The Vet-LIRN-CVM-FDA project’s mission aims to protect human and animal health by coordinating a network of veterinary diagnostic laboratories, conducting WGS of selected *Salmonella* isolates submitted by veterinary diagnostic laboratories nationwide, and tracking AMR genetic determinants associated with the use of veterinary drugs in different animal species (Food and Drug Administration, 2025). Out of the 33 *Salmonella* isolates submitted by BADDL for WGS, the Vet-LIRN-CVM-FDA project selected 12 isolates for WGS. Raw short reads of WGS data of these 12 isolates were available in the Sequence Read Archive (SRA) database of the National Center for Biotechnology Information (NCBI) under BioProject number PRJNA503850 (Date accessed September 1st, 2025) (Table 1). Furthermore, eight fecal samples from apparently healthy animals were freshly collected between 1 January 2025 and 30 June 2025 and processed for *Salmonella* isolation; during this period, one isolate each originated from an apparently healthy captive reptile, and a wild reptile was also included in the study. WGS of these two isolates was performed at SeqCenter (Pittsburgh, Pennsylvania, United States). The raw sequence data were deposited in the NCBI under BioProject number PRJNA 1331838 (Table 1).

**Table 1:** Details of the *Salmonella enterica* isolates analyzed for AMR and VF gene profiles.

No	Sample ID	Year of isolation	County	Host scientific name	Reptile type	Infection type	*Salmonella* species	Sub species	Serovar	SRA number in NCBI	Whole Genome size (bp)
1	SAL-18-VL-NY-FL-0020	2018	Lee	*Trachemys scripta*	Turtle	Gastrointestinal	*S. enterica*	*enterica*	Newport	SRR9016872	4,751,558
2	SAL-19-VL-NY-FL-0028	2019	Orange	*Alligator mississippiensis*	Allegator	Gastrointestinal	*S. enterica*	*enterica*	Oranienburg	SRR12401726	4,676,576
3	SAL-20-VL-NY-FL-0027	2020	Osceola	*Gopherus polyphemus*	Tortoise	Gastrointestinal	*S. enterica*	*enterica*	Oranienburg	SRR15357284	4,570,121
4	SAL-21-VL-NY-FL-0020	2021	Osceola	*Astrochelys radiata*	Tortoise	Gastrointestinal	*S. enterica*	*enterica*	Saintpaul	SRR23611336	4,706,297
5	SAL-20-VL-NY-FL-0013	2020	Osceola	*Crotalus mitchellii*	Snake	Gastrointestinal	*S. enterica*	*enterica*	III 38: (k): z35	SRR12991094	5,344,592
6	SAL-21-VL-NY-FL-0013	2021	Santa Rosa	*Python regius*	Snake	Gastrointestinal	*S. enterica*	*enterica*	lll_35: k:z53	SRR23602822	5,850,977
7	SAL-23-VL-NY-FL-0008	2023	Osceola	*Micrurus fulvius*	Snake	Gastrointestinal	*S. enterica*	*enterica*	III_57: k:e,n,x,z15	SRR30345702	4,715,185
8	SAL-20-VL-NY-FL-0001	2020	Alachua	*Python curtus*	Snake	Systemic	*S. enterica*	*houtene*	-	SRR12739107	4,585,827
9	SAL-21-VL-NY-FL-0021	2021	Alachua	*Elaphe obsoleta*	Snake	Systemic	*S. enterica*	*enterica*	III_50: r:z	SRR23611333	5,741,706
10	SAL-23-VL-NY-FL-0006	2023	Charlotte	*Cuora trifasciata*	Turtle	Systemic	*S. enterica*	diarizonae	III_50: k:z	SRR30345702	4,715,185
11	SAL-24-VL-NY-FL-0008	2024	Osceola	*Micrurus fulvius*	Snake	Systemic	*S. enterica*	*enterica*	Newport	SRR31882313	6,044,941
12	SAL-24-VL-NY-FL-0012	2024	Lee	*Chelonoidis niger*	Tortoise	Systemic	*S. enterica*	*enterica*	Rubislaw	SRR31882308	4,565,019
13	SAL-25-FL-0015	2025	Osceola	*Micrurus fulvius*	Snake	Apparently healthy	*S. enterica*	*arizonae*	40:z36:-	SRR35555236	4,736,437
14	SAL-25 W-FL-0001	2025	Lake	*Anolis sagrei*	Lizard	Apparently healthy	*S. enterica*	*enterica*	Gaminara	SRR35555235	5,277,599

SRA data of 12 isolates obtained from the NCBI during 2018 to 2024, as well as paired raw short read and single read libraries obtained from SeqCenter for the two *Salmonella* isolates in 2025, were uploaded into the Bacterial and Viral Resource Center (BVBRC) information system for genome assembly, annotation, and comprehensive genome analysis (Olson et al., 2023). Genome assembly and annotation were performed using SPAdes (V0.5.1) (Wick et al., 2017) and the RAST toolkit (RASTtk) (Brettin et al., 2015), respectively. The special genes homologous to AMR and VF genes were characterized using the Pathosystems Resource Integration Center (PATRIC) (Wattam et al., 2017) and the Virulence Factor Database (VFDB) (Chen et al., 2005), respectively. To identify antimicrobial groups, AMR genes were categorized using the PATRIC database. Heat maps representing protein families for both AMR and susceptible isolates were generated using the BVBRC Comparative Systems tool, while the BVBRC Proteome Comparison Tool was used to determine protein similarity percentages across the 10 *Salmonella* isolates (Olson et al., 2023). The multiple sequence alignment (MSA) tool in BVBRC was used to detect single-nucleotide polymorphism (SNP) in identified protein families from both AMR and susceptible isolates (Olson et al., 2023). Furthermore, VF genes related to adherence, immune evasion, invasion, toxin production, stress response, and Type III secretion systems were categorically identified using VFDB (Chen et al., 2005). To visualize the distribution and presence of these genes, heatmaps were generated in Python using libraries: Seaborn (Waskom, 2021) for visualization and Matplotlib (Hunter, 2007) for customization. These findings were subsequently analyzed within the context of public health risks. Data preparation and tabulation were performed using Microsoft® Excel® (V 2402).

The presence of plasmid sequences in the 14 WGS *Salmonella* isolates was analyzed using PlasmidFinder (V 2.1.6) (Carattoli and Hasman, 2019). Previously assembled contigs by BVBRC from 14 isolates were analyzed using PlasmidFinder (V 2.1.6, installed on the Galaxy server V 23.2.2) to identify plasmid sequences in the assembled contigs with 95% identity and 80% query coverage.

### 2.4 Plasmid DNA sequencing and analysis

Pure cultures of four *Salmonella* isolates listed in Table 2 were cultured in 40 mL LB broth tubes and incubated at 37 ± 2 °C for 12 h. The selection of isolates was based on phenotypic AMR and/or susceptibility to cefazolin (FAZ) and gentamicin (GEN), health status of the host (diseased or healthy), and host environment (Captive or wild). Following incubation, plasmid DNA was extracted using the QIAGEN® Plasmid Midi Kit (QIAGEN, Germantown, Maryland, United States) according to the manufacturer’s protocol. Extracted plasmid DNA was submitted for 300 Mbp-long-read sequencing at the SeqCenter, employing Oxford Nanopore Technology (ONT) (Table 2). The residual adaptor sequence of raw reads obtained from the ONT was trimmed using Porochop open-source software (V 0.2.4)^1^ with default parameters. *De novo* genome assembly was performed using Flye (V 2.9.2) (Kolmogorov et al., 2019). Additional Flye options initiated disjoining assembly with the longest reads, targeting approximately 50x assembly coverage metrics and assuming an estimated genome size of 6 Mb. Assembled contigs were evaluated for circularization via Circlator (V 1.5.1) (Hunt et al., 2015). Genome annotation was performed using Bakta (V 1.8.1), and assembly quality was assessed with QUAST (V5.2.0) (Schwengers et al., 2021; Gurevich et al., 2013). PlasmidHunter (V 1.4, installed on the Galaxy server V 23.2.2) was used to identify plasmid sequences among assembled contigs from four *Salmonella* isolates (Table 2; Tian et al., 2024). The contigs predicted by PlasmidFinder as plasmid-derived from four *Salmonella isolates* were separately input into the Proksee (V 20260110) (Grant et al., 2023) to identify open reading frames (ORFs), followed by functional annotation using Bakta V 1.1.0 (Schwengers et al., 2021). AMR genes were identified by the Comprehensive Antibiotic Resistance Database (CARD Resistance Gene Identifier RGI) (V 1.3.1) (Alcock et al., 2023), whereas Mobile Orthologous (MobileOG) groups were characterized by the MobileOG database (Brown et al., 2022; Table 3).

**Table 2:** Phenotypic resistance of the studied *Salmonella enterica* isolates.

Isolate name	Phenotypic resistance observed in antibiotic sensitivity testing
SAL-18-VL-NY-FL-0020	Susceptible to all tested antibiotics
SAL-19-VL-NY-FL-0028	Amikacin, Cefazolin, Cephalexin, Gentamycin, and Ampicillin
SAL-20-VL-NY-FL-0027	Amikacin, Cefazolin, Cephalexin, Gentamycin, and Ampicillin
SAL-21-VL-NY-FL-0020	Amikacin, Cefazolin, Cephalexin, Gentamycin, and Ampicillin
SAL-20-VL-NY-FL-0013	Amikacin, Cefazolin, Cephalexin, and Ampicillin
SAL-21-VL-NY-FL-0013	Amikacin, Cefazolin, Cephalexin, Gentamycin, Doxycycline, and Tetracycline
SAL-23-VL-NY-FL-0008	Susceptible to all tested antibiotics
SAL-20-VL-NY-FL-0001	Amikacin, Cefazolin, Cephalexin, Gentamycin, and Ampicillin
SAL-21-VL-NY-FL-0021	Amikacin, Cefazolin, Cephalexin, Gentamycin, Ampicillin, and Augmentin
SAL-23-VL-NY-FL-0006	Susceptible to all tested antibiotics
SAL-24-VL-NY-FL-0008	Susceptible to all tested antibiotics
SAL-24-VL-NY-FL-0012	Susceptible to all tested antibiotics
SAL-25-FL-0015	Amikacin, Cefazolin, Cephalexin, Gentamycin, and Ampicillin
SAL-25 W-FL-0001	Amikacin, Cefazolin, Cephalexin, Gentamycin, and Ampicillin

**Table 3:** Details of the plasmid DNA analysis.

Isolate	Number of assembled contigs	Number of predicted plasmids by Plasmid Hunter	Number of CDS	Antimicrobial resistance genes identified by CARD RGI	Number of Mobile elements identified by mobileOG -db				
					IE	*p*	RRR	STD	T
SAL-20-VL-NY-FL-0013	66	21	1,092	0	2	9	0	0	0
SAL-21-VL-NY-FL-0013	93	54	1833	tetA	107	43	39	50	107
SAL-24-VL-NY-FL-0008	93	54	1,075	0	2	4	2	3	10
SAL-25 W-FL-0001	26	11	987	0	7	45	4	10	69

IE, Integration/Excision; P, Phage; RRR-Replication/recombination/repair; STD, Stability/Transfer/defense; T, Transfer; CDS, Coding strands; mobileOD-db, Mobile Orthologous Groups Database; CARD RGI, Comprehensive antibiotic resistance database resistance gene identifier; tetA, Tetracycline A gene.

### 2.5 Transcriptomic analysis

*Salmonella* isolates (listed in Table 2) were cultured in sub-minimum inhibitory concentrations (sub-MIC) of 2 μg/mL gentamicin (GEN) and 8 μg/mL Cefazolin (FAZ) in LB broth for 6 h at 37 ± 2 °C before RNA sequencing. The selection of isolates was targeted to conduct an observational transcriptomic study, considering the representation of isolates with phenotypic AMR and/or susceptibility to cefazolin (FAZ) and gentamicin (GEN), health status of the host (disease or healthy), and host environment (captive or wild). All four isolates were revived from glycerol stocks stored at −80 °C, transferred onto LB agar plates (Fisher Bioreagents, Waltham, Massachusetts, United States), and incubated overnight at 37 ± 2 °C. Following incubation, pure isolates confirmed by the MALDI Biotyper Sirius system were transferred to LB broth (Fisher Bioreagents, Waltham, Massachusetts, United States) and incubated overnight at 37 ± 2 °C. A series of two-fold dilutions of gentamicin (GEN; MP Biomedicals, Solon, Ohio, United States) and cefazolin (FAZ; ThermoFisher Scientific, Waltham, Massachusetts, USA) was prepared in 10 mL of LB broth. These ranges were based on the concentrations provided in the Sensititre™ Companion Animal Gram-Negative COMPGN1F Vet AST Plate (ThermoFisher Scientific, Cleveland, Ohio, United States). Overnight *Salmonella* cultures were inoculated into the antibiotic-amended broth and an antibiotic-free negative control; these cultures were then incubated for 6 h to identify the optimal sub-MIC point for RNA extraction. Tubes showing turbidity equivalent to the negative control were selected for downstream RNA processing.

For transcriptome analysis, the four isolates were cultured in 40 mL LB broth containing GEN (2 μg/mL) and FAZ (8 μg/mL) for 6 h at 37° ± 2 °C, and the tubes were centrifuged and washed twice in phosphate buffered saline (PBS) at 10000 x g for 20 min at 4 ± 2 °C to obtain the cell pellet. Following the final centrifugation step, the supernatant was discarded, and the cell pellet was preserved using RNAlater™ (Carlsbad, California, United States) and submitted to SeqCenter for RNA extraction and sequencing. RNA extraction was performed using the ZymoBIOMICS™ quick-RNA miniprep kit (Zymo Research, Irvine, California, USA) according to the recommended protocol. Following extraction, samples were treated with DNase (Invitrogen™, Carlsbad, California, USA) to remove residual genomic DNA. Library preparation was performed using Illumina Stranded mRNA Prep (Illumina, San Diego, California, USA), and sequencing was conducted on an Illumina NovaSeq X plus, generating 150 bp paired-end reads. Demultiplexing, quality control, and adaptor trimming were performed using bcl-convert V 4.2.4 (Illumina, San Diego, California, USA). Paired raw reads were deposited in the NCBI under BioProject number PRJNA1425458. Read mapping was conducted using hisat2 (V 2.2.0) (Kim et al., 2019). Gene-level read quantification for functional analysis was performed using featureCounts (V2.0.1) from the Surbread package (Liao et al., 2014). Before expression analysis, raw quantified counts were imported into R (V 4.0.2) (R Core Team, 2020) and normalized using edgeR (V 1.14.5) trimmed mean of M-values (TMM) algorithm. Differential expression analysis between treatment and control groups was performed using edgeR’s exact test for negative binomial count data with an estimated dispersion value of 0.1 (Robinson et al., 2010). Subsequently, the subset of differentially expressed genes was identified based on fold changes (Log_2_ > 1) and *p*-values < 0.05. Normalized counts per million (CPM) values for differentially expressed genes (DEG) were used to create heatmaps. Further, the DEG list was uploaded into Microsoft® Excel® V 2402 for tabulation and further functional clearing. The gene functions containing the terms “Multidrug resistance,” “Multidrug efflux,” “Multidrug transporter,” and “Resistance” associated with specific antimicrobial names were extracted to a new worksheet for further analysis. Functional annotation and AMR gene identification were performed using the CARD Resistance Gene Identifier (RGI) (V 1.3.1) (Alcock et al., 2023), UniProt (The UniProt Consortium, 2017), and InterPro database (Paysan-Lafosse et al., 2023).

## 3 Results

### 3.1 Description of *Salmonella* isolates and epidemiology of *Salmonella* in reptiles

*Salmonella* testing was conducted by BADDL for two main purposes: disease diagnosis and regulatory export. Of the total, 142 cultures (69.15%) were performed for routine microbiology diagnostics, 35 (17.41%) for necropsy-related diagnostics, and 24 (11.94%) cultures for regulatory purposes. Thus, 201 case accessions were received during the time period with *Salmonella* culture test requests. A total of 257 reptile samples were tested for *Salmonella* culture under these case accessions. *Salmonella* was recovered from 33 case accessions which included 45 reptile samples implying a 16.41% case positivity rate and a 17.51% sample positivity rate.

Of the 33 *Salmonella* isolates, 6 were serogrouped as group C, and 2 isolates were grouped as group B (Supplementary Table 1). The remaining isolates were either not subjected to serogrouping or yielded inconclusive results (Supplementary Table 1). Of the 33 isolates, 23 were serotyped. Of the 23, 17 isolates belong to *Salmonella enterica* subspecies *enterica*, four to subspecies *diarizonae*, and one each to subspecies *arizonae* and *houtine*. Known serotypes among the isolates included *Salmonella* Braenderrup, Sandiego, Newport, Oranienburg, Saintpul, and Kisarawa, and others were unknown serovars (Supplementary Table 1). Serotyping was not performed for the remaining 10 isolates (Supplementary Table 1). Reptiles belong to three taxonomic orders, namely Squamata, which consists of snakes, iguanas, lizards, geckos, and dragons, Testudines, which consists of tortoises and turtles, and Crocodilia, which consists of alligators. The highest positivity rate of 34.21% was recorded in the order Squamata, followed by 12.2% in Testudines and Crocodilia.

### 3.2 Antimicrobial resistance

**Figure 1:**
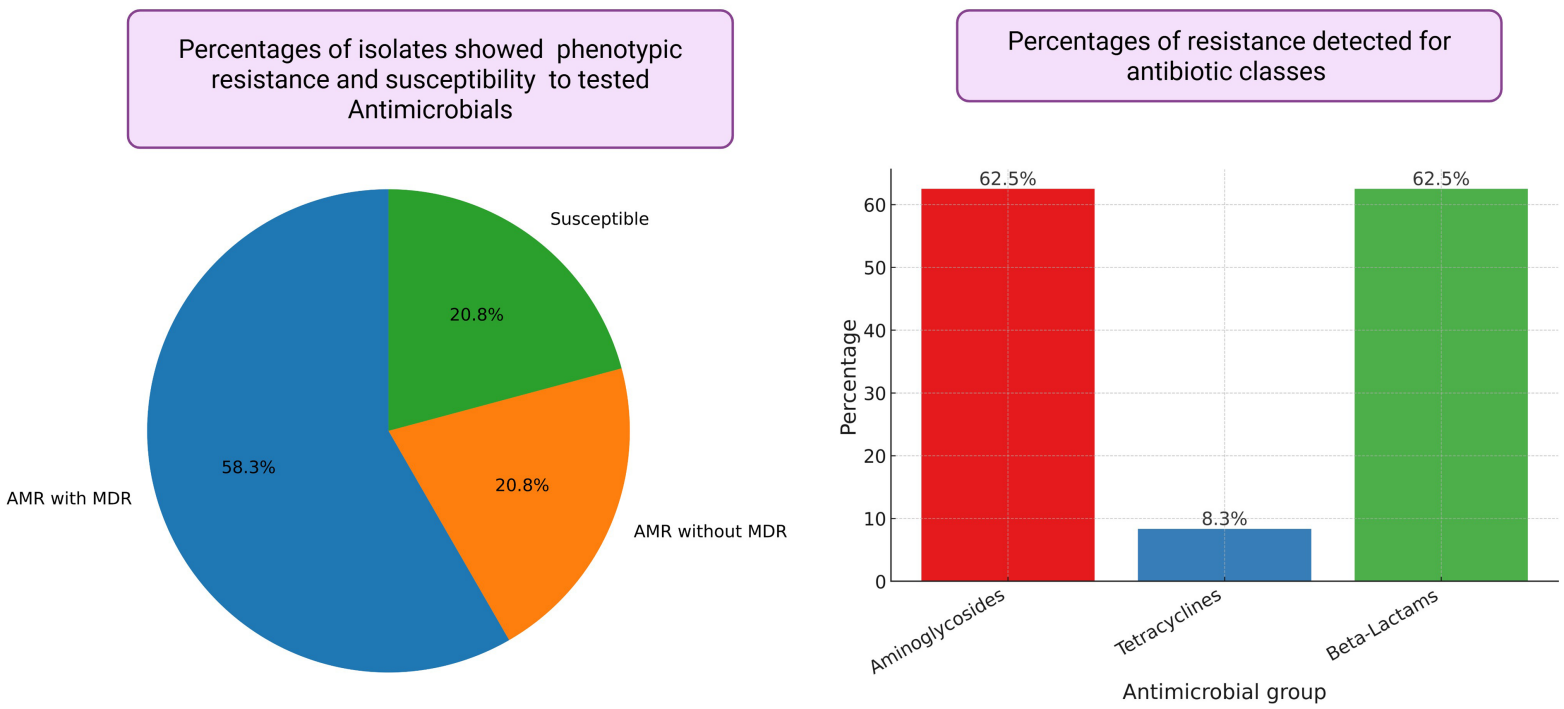
Phenotypic antimicrobial susceptible, antimicrobial resistance (AMR), multi drug resistance (MDR) profiles of non typhoidal *Salmonella* isolates used in this study.

**Figure 2:**
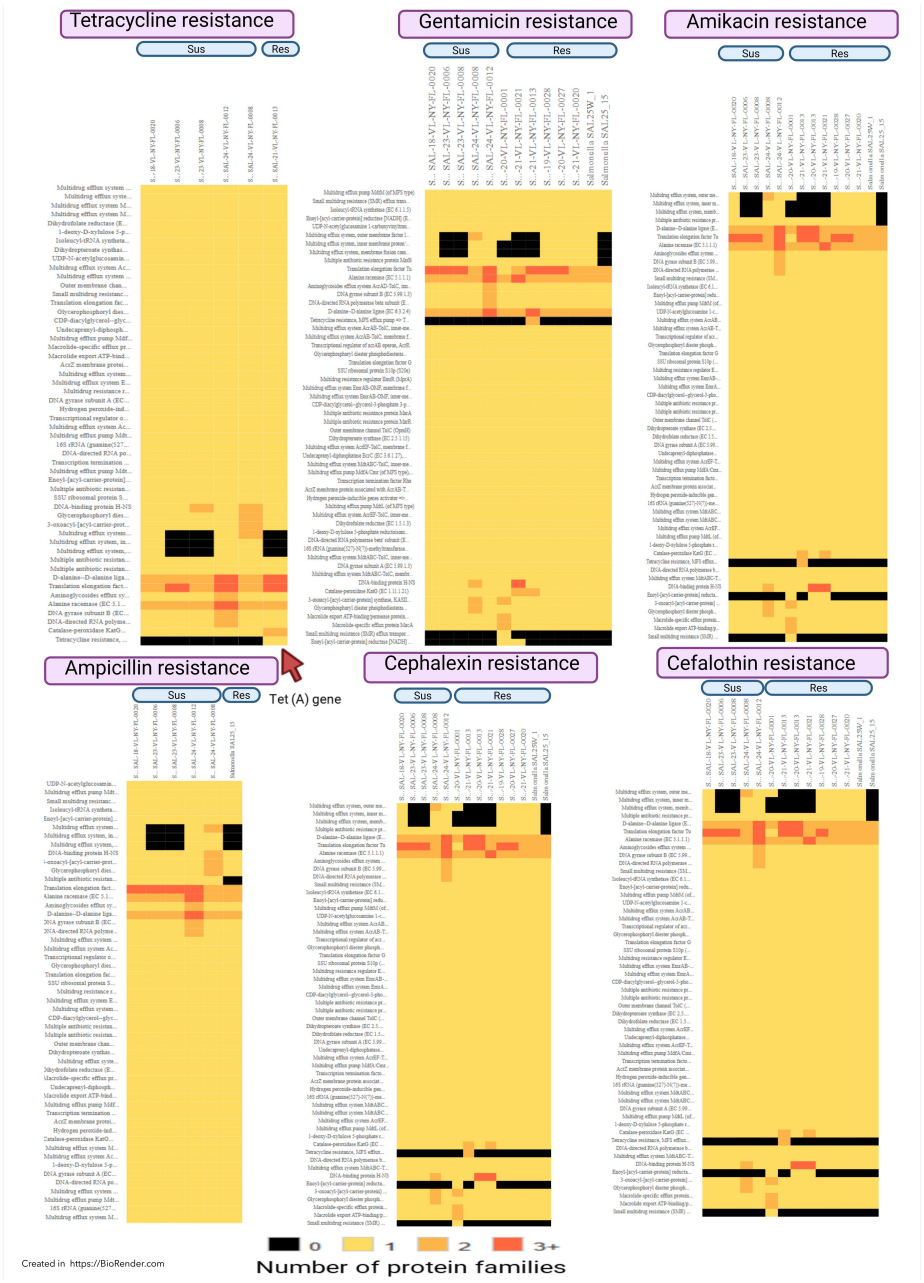
Heat maps of the selected antimicrobial resistance protein families of antimicrobial resistance (Res) and susceptible (Sus) non typhoidal *Salmonella enterica* from 2018 to 2025.

**Figure 3:**
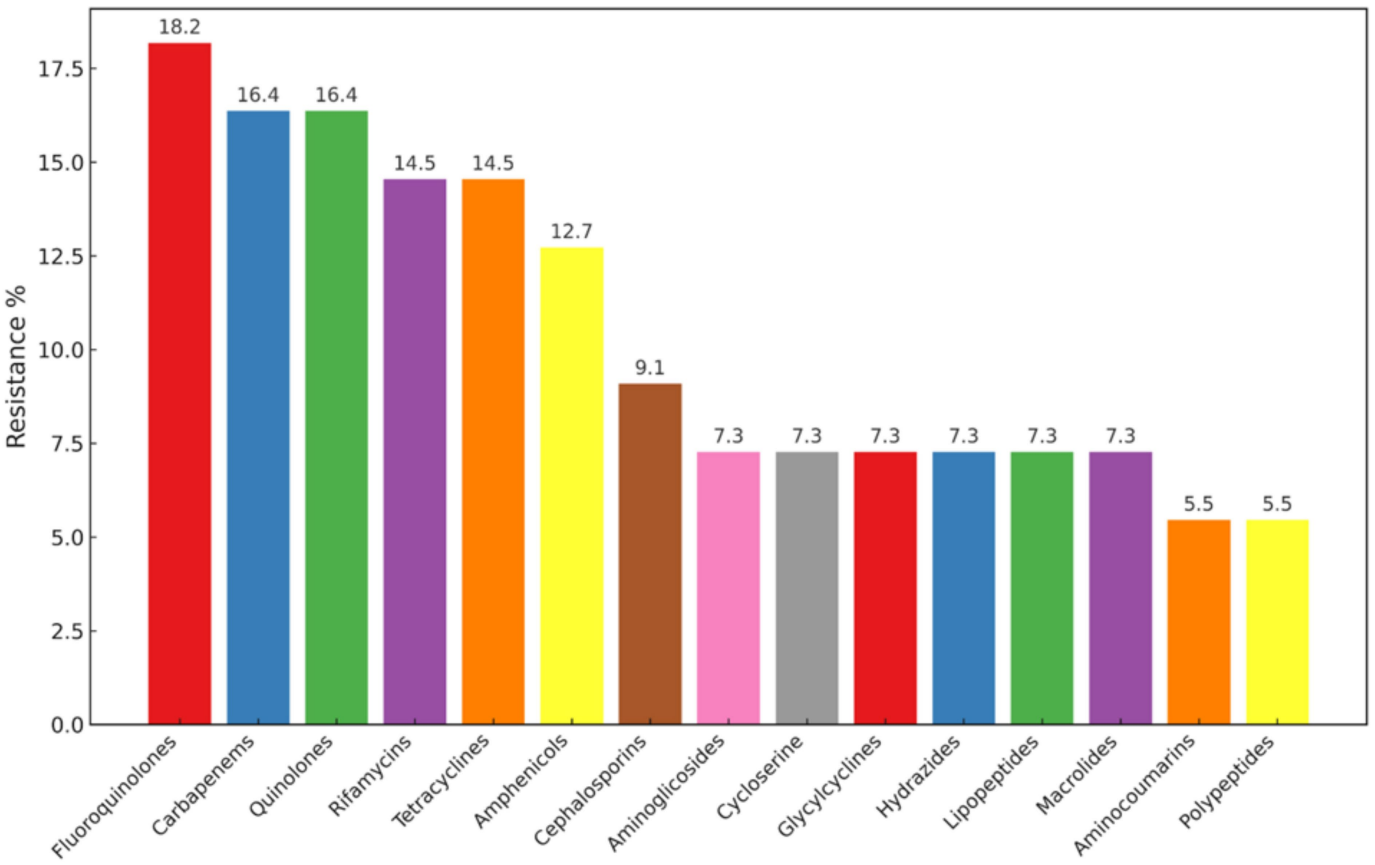
Percentages of AMR genes detected against antimicrobial groups.

**Figure 4:**
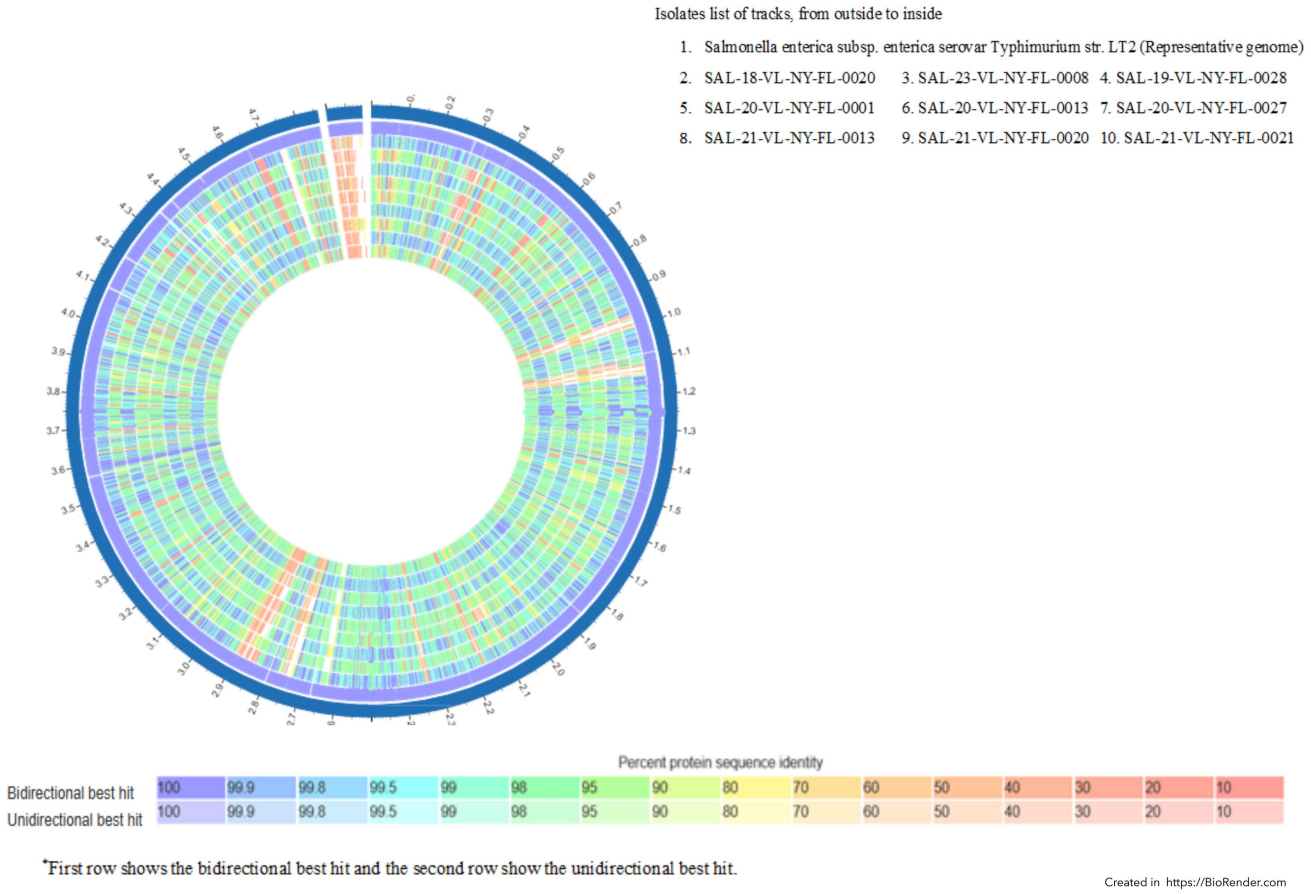
Proteome comparison of non-typhoidal *Salmonella enterica* isolates originated from reptiles from 2018 to 2023.

Of the 24 isolates subjected to AST, 14 were resistant to antibiotics, with resistance to beta-lactams (62.5%), aminoglycosides (62.5%), and tetracyclines (8.3%) (Figure 1). Of the 24 isolates that underwent AST, 14 showed AMR, including 58.3% with MDR. Five isolates showed AMR without MDR (20.8%), and five isolates (20.8%) were susceptible to the tested antibiotics (Figure 1). Phenotypic resistance was observed for ampicillin, augmentin, cephalexin, and cefazolin under beta-lactams; amikacin and gentamicin under aminoglycosides; and tetracycline and doxycycline under tetracycline antibiotic groups. Of all *Salmonella enterica* isolated from reptiles by BADDL during the study period, 14 were analyzed by whole-genome sequencing, which revealed 55 AMR genes homologous to those in the PATRIK database (Supplementary Table 2; Figure 2). Of the 55 AMR genes, 10 genes showed resistance to fluoroquinolones (18.18%), 9 genes resistance to carbapenems and quinolones (16.36%), 8 genes resistance to tetracyclines and rifamycin (14.54%), 7 genes resistance to amphenicols (12.72%), 5 genes resistance to cephalosporins (9.09%), and 4 genes resistance to aminoglycosides (7.27%) (Figure 3). The protein family related *tet*A gene was detected only in SAL-21-VL-NY-FL-0013 among the 14 isolates, which showed phenotypic resistance to tetracycline and doxycycline (Table 2; Figure 2). Proteomic comparison of 10 isolates revealed that similarity percentages for each protein ranged from 10 to 100% (Figure 4). MSA of amino acid sequences from the 55 proteins between antimicrobial-resistant and susceptible isolates in this study revealed that amino acid changes in each protein did not make that particular isolate resistant or susceptible to the antimicrobials.

### 3.3 Whole genome analysis and virulence factor genes

**Figure 5:**
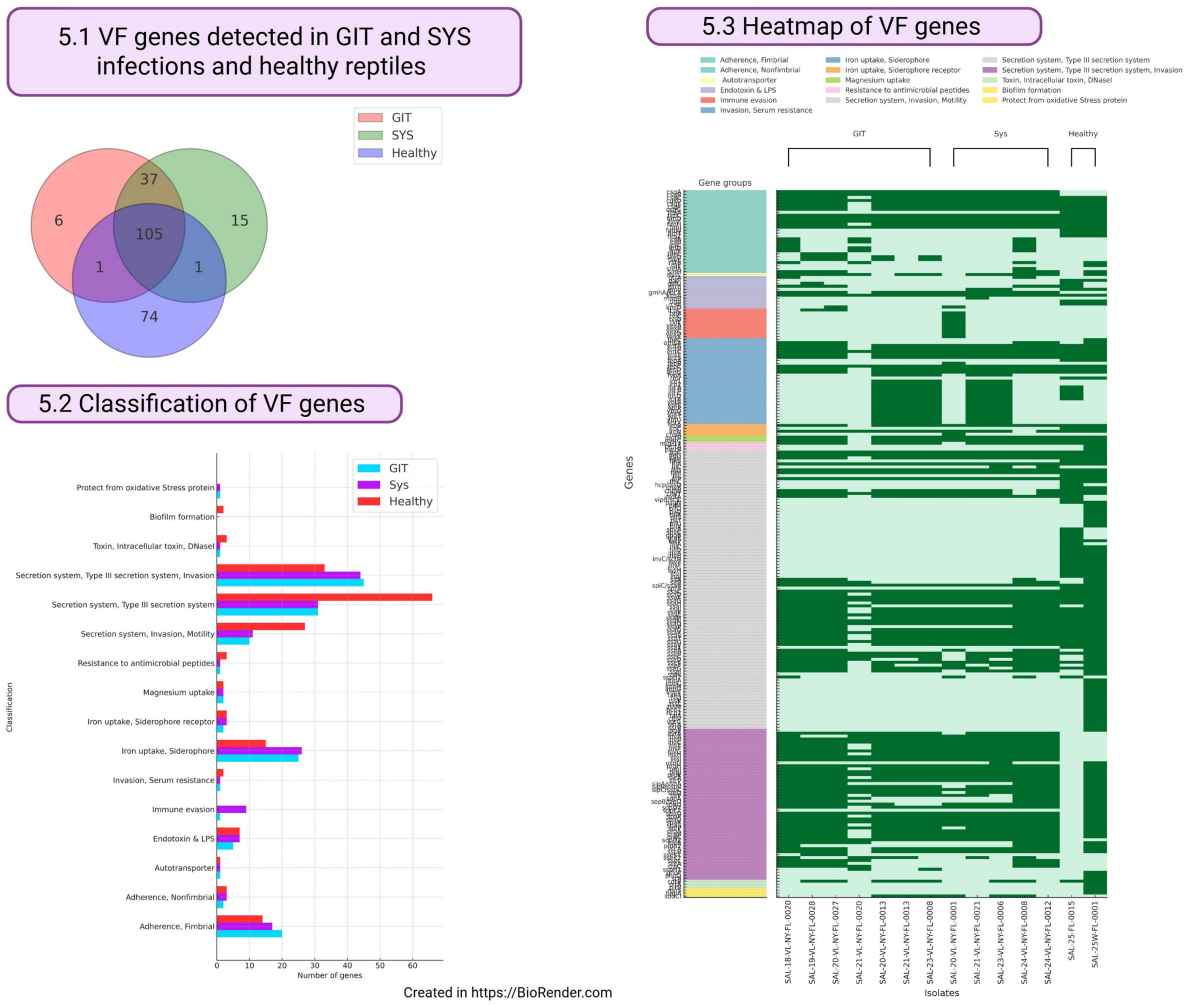
Virulence factor (VF) genes detected in *Salmonella enterica* isolates originated from reptiles.

The size of the whole genomes of the 14 *Salmonella* isolates subjected to WGS varies from 4,565,019 to 6,044,941 base pairs (Table 1). By conducting WGS analysis of 14 isolates (Table 1), a total of 239 VF genes were identified using the VFDB (Chen et al., 2005). These genes were found across seven GIT samples, five systemic infections, and two healthy reptiles. Specifically, 148 VF genes of *Salmonella* were identified in GITsamples, 158 were identified in systemic infections, and 181 were identified in healthy reptiles. Of the 105 VF genes, 37 were common to GIT and systemic infections and healthy animals; 1 VF gene was common to both GIT infections and healthy animals; and systemic and healthy animals (Figure 5; Supplementary Table 3). A total of 74 VF genes were unique to isolates from healthy animals, while 15 and 6 genes were exclusively associated with systemic and gastrointestinal (GIT) infections, respectively. (Figure 5). Identified genes were clustered by main function (Figure 5). Regardless of infection status or animal health, the highest number of genes were identified in the type III secretory system, invasion, motility, iron uptake, siderophore, fimbrial adherence, endotoxin, and lipopolysaccharides (Figure 5). As expected, the highest number of immune evasion VF genes was identified in *Salmonella* isolates involved in systemic infections, while the highest number of fimbrial adherence genes was observed in *Salmonella* isolates involved in GIT infections (Figure 5).

Plasmid analysis using WGS data revealed the presence of the plasmid replication initiation protein (*repA*) gene in isolates SAL-18-VL-NY-FL-0020, SAL-20-VL-NY-FL-0027, and SAL-24-VL-NY-FL-0008 with 100, 99.81, and 99.81% nucleotide identity, respectively.

### 3.4 Plasmid DNA analysis

Plasmid DNA analysis revealed that the plasmid of isolate SAL-21-VL-NY-FL-0013 carries the *tetA* gene (Table 3) and that it confers phenotypic resistance to tetracycline and doxycycline (Table 2). Mobile genetic elements responsible for integration/excision, phage, replication/recombination/repair, stability/transfer/defense, and transfer were identified in the plasmids recovered from the four *Salmonella enterica* isolates listed in Table 3.

### 3.5 Transcriptomic analysis

**Figure 6:**
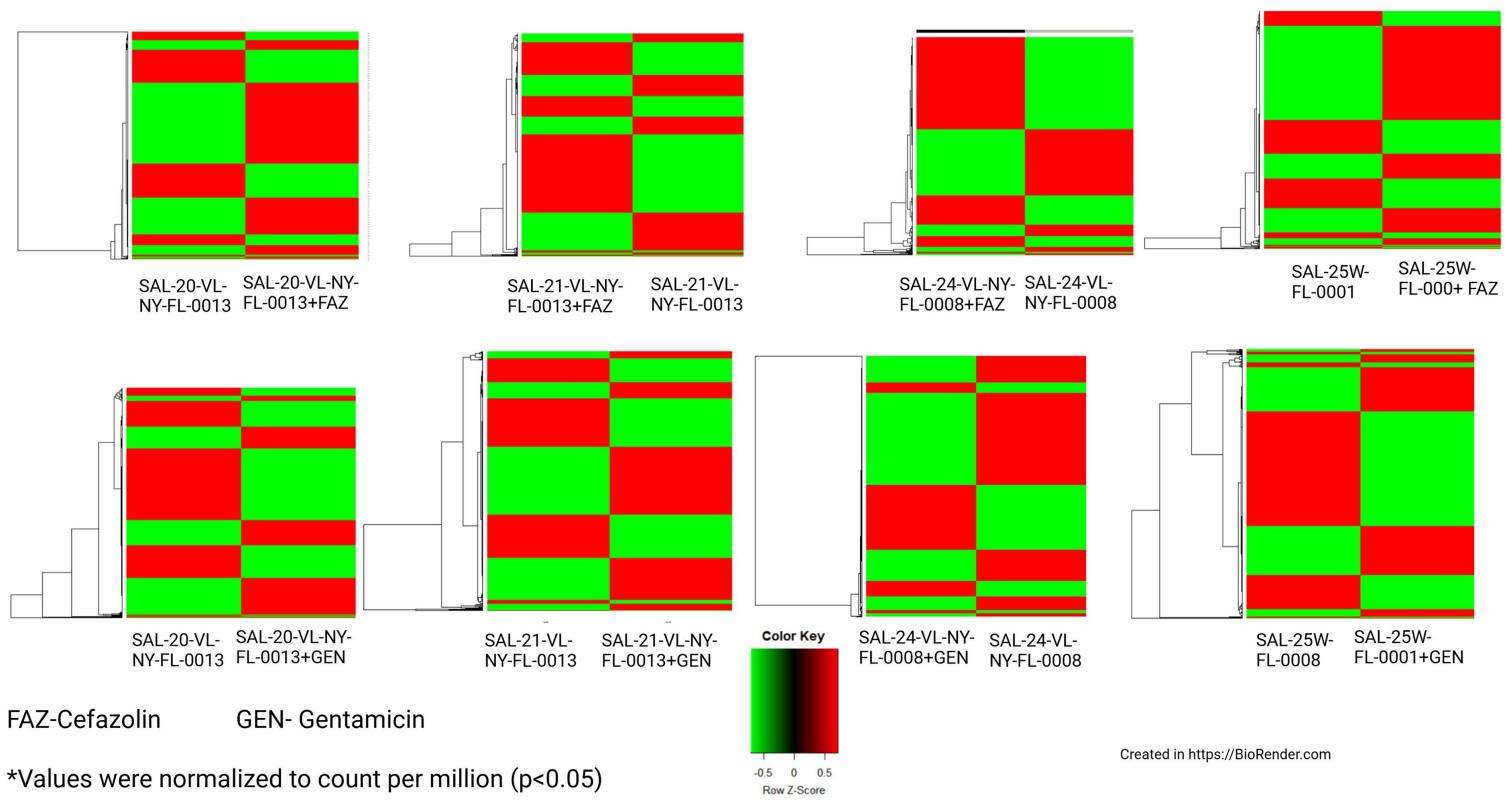
Heatmaps of the differentially expressed gene’s in selected *Salmonella enterica* isolates grown with and without antimicrobials*.

RNA analysis of selected *Salmonella enterica* isolates from captive and wild reptiles, grown at sub-MICs of FAZ and GEN, revealed differential expression of AMR genes and their relationship to phenotypic AMR profiles (Figure 6; Tables 4, 5). Transcriptome profiles of SAL-20-VL-NY-FL-0013, SAL-21-VL-NY-FL-0013, SAL-24-VL-NY-FL-0008, and SAL-25 W-FL-0001 grown in the presence of sub-MIC of FAZ exhibited 772, 834, 696, and 506 differentially expressed genes (DEGs), respectively (Log_2_ fold change > 1 and *p* < 0.05). Of the DEGs from the four isolates described, 19, 17, 10, and 7 genes were associated with AMR, respectively (Table 4). Among the DEGs relevant to AMR, 17, 12, 8, and 6 genes, in isolates SAL-20-VL-NY-FL-0013, SAL-21-VL-NY-FL-0013, SAL-24-VL-NY-FL-0008, and SAL-25 W-FL-0001, respectively, which corresponded to AMR genes previously identified through WGS (Table 4). Beyond the previously identified AMR determinants, transcriptomic analysis uncovered additional AMR-associated transcripts—specifically 2, 5, 2, and 1 AMR-related genes, across the respective isolates—that were not captured in the initial genomic screening (Table 4). Functional categorization via the InterPro database (Paysan-Lafosse et al., 2023) mapped these DEGs to critical protein families, including MDR systems, efflux pumps, and specialized transporters (*p* < 0.05; Table 4). The isolate SAL-20-VL-NY-FL-0013, which showed phenotypic resistance to amikacin, cefazolin, and cephalexin when grown in the presence of FAZ, showed upregulation of the Major Facilitator Superfamily (MFS) profile domain-containing protein and MDR efflux pump genes by 3.358 and 2.081-Log_2_ folds, respectively (Table 4). Furthermore, the isolate SAL-25 W-FL-0001, resistant to amikacin, cefazolin, cephalexin, and gentamicin, showed the upregulation of MFS profile domain-containing protein by 12.369-Log_2_ folds (Table 4). The isolate SAL-21-VL-NY-FL-0013, resistant to amikacin, cefazolin, cephalexin, doxycycline, tetracycline, and gentamicin, showed upregulation of two genes, namely MFS profile domain-containing protein and aminoglycoside N (6’)-acetyltransferase type 1 by 10.856- and 8.763-Log_2_ folds, respectively. However, the same isolate showed downregulation of three genes, namely, the ABC-type multidrug/protein/lipid transport system, undecaprenyl-diphosphatase, and p-hydroxybenzoic acid efflux pump subunit gene *aaeA* by 1.481-, 1.952-, and 2.328-Log2 fold, respectively (Table 4). The FAZ susceptible isolate, SAL-24-VL-NY-FL-0008, showed the downregulation of *aaeA* by 1.539-Log_2_ fold and upregulation of MFS profile domain-containing protein by 4.134-Log_2_ fold (Table 4).

**Table 4:** Details of the differentially expressed (*p* < 0.05) antimicrobial-related genes of *Salmonella enterica* isolates grown with cefazoline (FAZ) versus the absence of cefazoline.

Isolates	SAL-20-VL-NY-FL-0013	SAL-21-VL-NY-FL-0013	SAL-24-VL-NY-FL-0008	SAL-25 W-FL-0001
Phenotypic resistance	Amikacin, Cefazolin, Cephalexin	Amikacin, Cefazolin, Cephalexin, Doxycycline, Tetracycline, Gentamycin	–	Amikacin, Cefazolin, Cephalexin, Gentamycin
No. of total genes	772	834	696	506
No. of genes related to AMR	19	17	10	07
No of genes concurring with WGS analysis	17	12	08	06
No of genes newly identified	02	05	02	01
Locus tag and gene name* (UniProt), protein name and protein family (InterPro), and Log_2_ expression value of newly identified genes	STM1545, Major facilitator superfamily (MFS) profile domain-containing protein, Major facilitator superfamily (MFS), and 3.358˄ STM1442, ydhJ, Multidrug resistance efflux pump, Membrane Fusion Protein (MFP), and 2.081 ˄	STM1545, Major facilitator superfamily (MFS) profile domain-containing protein, Major facilitator superfamily (MFS), and 10.856˄ STM1619, Aminoglycoside N (6′)-acetyltransferase type 1, Bacterial Acetyltransferase, and 8.763˄ STM2263, yojl, ABC-type multidrug/protein/lipid transport system, Type 1 protein exporter, and 1.481˅ STM3205, bacA, Undecaprenyl-diphosphatase, Undecaprenyl-diphosphatase UppP, and 1.952˅ STM3365, aae, p-hydroxybenzoic acid efflux pump subunit AaeA, Membrane Fusion Protein (MFP), and 2.328˅	STM1545, Major facilitator superfamily (MFS) profile domain-containing protein, Major facilitator superfamily (MFS), and 4.134˄ STM3365, aae, p-hydroxybenzoic acid efflux pump subunit AaeA, Membrane Fusion Protein (MFP), and 1.539˅	STM1545, Major facilitator superfamily (MFS) profile domain-containing protein, Major facilitator superfamily (MFS), and 12.369 ˄

**Table 5:** Details of the differentially expressed (*p* < 0.05) antimicrobial-related genes of *Salmonella enterica* isolates grown with gentamycin (GEN) versus the absence of gentamycin.

Isolates	SAL-20-VL-NY-FL-0013	SAL-21-VL-NY-FL-0013	SAL-24-VL-NY-FL-0008	SAL-25 W-FL-0001
Phenotypic resistance	Amikacin, Cefazolin, Cephalexin	Amikacin, Cefazolin, Cephalexin, Doxycycline, Tetracycline, Gentamycin	–	Amikacin, Cefazolin, Cephalexin, Gentamycin
No. of total genes	721	636	1,155	440
No. of genes related to AMR	17	10	17	03
No of genes concurring with WGS analysis	13	07	10	02
No of genes newly identified	04	03	07	01
Locus tag and gene name* (UniProt), protein name, protein family (InterPro), and Log_2_ expression value of newly identified genes	STM1545, Major facilitator superfamily (MFS) profile domain-containing protein, Major facilitator superfamily (MFS), and 7.903˅ STM1748, ychE, Inner membrane protein, Multiple antibiotic resistance (MarC)-related, and 2.542˄ STM3365, aae, p-hydroxybenzoic acid efflux pump subunit AaeA, Membrane Fusion Protein (MFP), and 1.624˄ STM3955, rarD, Protein RarD, RarD protein, and 3.680˄	STM3365, aae, p-hydroxybenzoic acid efflux pump subunit AaeA, Membrane Fusion Protein (MFP), and 2.807˅ STM0257, Major facilitator superfamily (MFS) profile domain-containing protein, Major Facilitator Superfamily Efflux Transporters, and 3.503˅ STM1122, ycdC, HTH tetR-type domain-containing protein, HTH-type, TetR-like transcriptional regulator, and 2.375˅	STM1545, Major facilitator superfamily (MFS) profile domain-containing protein, Major facilitator superfamily (MFS), and 1.938˅ STM0257, Major facilitator superfamily (MFS) profile domain-containing protein, Major Facilitator Superfamily Efflux Transporters, and 3.468˅ STM1575, HTH tetR-type domain-containing protein, HTH-type, TetR-like transcriptional regulator, and 4.027˄ STM1619, Aminoglycoside N (6’)-acetyltransferase type 1, Bacterial Acetyltransferase, and 8.556˅ STM3205, bacA, Undecaprenyl-diphosphatase, Undecaprenyl-diphosphatase UppP, and 1.399˅ STM2263, yojl, ABC-type multidrug/protein/lipid transport system, Type 1 protein exporter, and 1.583˄ STM3586, yhiH, ABC-type multidrug transport system, Ribosome-associated ATPase RbbA, and 1.438˅	STM1545, Major facilitator superfamily (MFS) profile domain-containing protein, Major facilitator superfamily (MFS), and 7.389˄

*Only the gene name that appears in the UniProt database has been used. The absence of the gene name indicates it is not present in the UniProt database. ˄Upregulated; ˅Downregulated.

Isolates SAL-20-VL-NY-FL-0013, SAL-21-VL-NY-FL-0013, SAL-24-VL-NY-FL-0008, and SAL-25 W-FL-0001 grown under gentamicin stress showed 721, 636, 1,155, and 440 DEGs (Log_2_ fold change > 1 and *p* < 0.05). Among those genes, 17, 10, 17, and 3 were associated with AMR (Table 5). The number of differentially expressed AMR genes concurring with WGS results in the aforesaid four isolates was 13, 07, 10, and 2, respectively. Transcriptomic analysis unmasked 4, 3, 7, and 1 novel AMR-related genes, respectively, that were not detected in the WGS analysis (Table 5). Isolate SAL-20-VL-NY-FL-0013, which exhibited resistance to amikacin, cefazolin, and cephalexin, showed downregulation of MFS profile domain-containing protein by 7.90-Log_2_ fold when grown in the presence of GEN. Conversely, the same isolate demonstrated upregulation of the inner membrane proteins, *aaeA*, and *rarD* genes by 2.5-, 1.6-, and 3.68-Log_2_ fold, respectively (Table 5). The isolate SAL-25 W-FL-0001, resistant to amikacin, cefazolin, cephalexin, and gentamicin, showed an upregulation of MFS profile domain-containing protein by 7.389-Log_2_ fold (Table 5) when grown in the presence of GEN. SAL-21-VL-NY-FL-0013, phenotypically resistant to amikacin, cefazolin, cephalexin, doxycycline, tetracycline, and gentamicin, exhibited downregulation of three genes encoding AaeA, an MFS profile domain-containing protein efflux transporter, and a helix-turn-helix (HTH) *tetR*-type domain-containing protein by 2.807, 3.503, and 2.375-Log_2_ folds, respectively (Table 5). Conversely, isolate SAL-24-VL-NY-FL-0008, which was susceptible to GEN, showed the downregulation of MFS profile domain-containing protein, MFS profile domain-containing protein efflux transporter, aminoglycoside N (6′)-acetyltransferase type 1, undecaprenyl-diphosphatase, and ABC-type multidrug transport system by 1.938-, 3.468-, 8.556-, 1.399-, and 1.438-Log_2_ folds, respectively. However, upregulation of genes encoding HTH TetR-type domain-containing protein by 4.027-Log_2_ folds and ABC-type multidrug/protein/lipid transport system by 1.583-Log_2_ folds was also observed in this isolate (Table 5).

## 4 Discussion

### 4.1 Host diversity of non-typhoidal *Salmonella* in reptiles

This study demonstrated a 15.42% prevalence of non-typhoidal *Salmonella* in captive reptiles during the study period, with the highest prevalence in the order Squamata compared to orders Testudines and Crocodilia. The high prevalence of *Salmonella* in predatory reptiles, relative to other reptiles, is due to the interplay of dietary exposure, stress-induced shifts in the gut microbiome, host permissiveness, early-life colonization, and captivity conditions (Paphitis et al., 2025).

A comprehensive meta-analysis conducted previously, the first of its kind, assessed the global prevalence of *Salmonella* in reptiles and key influencing factors by reviewing 179 studies (1986–2023) covering 23,411 reptiles from 56 countries. This study had 49.9% samples originating from the wild, and 48.4% originating from captive reptiles (zoos, pet shops, households). This study found results similar to those of the study, with a pooled prevalence of 30.4% (95% CI: 27.4–33.6%). Snakes had the highest prevalence at 63.1% (95%CI: 57.4–68.4%), followed by lizards at 33.6% (95%CI: 28.6–39.0%), turtles 11.2% (95%CI: 8.8–14.2%), and crocodiles 10.5% (95%CI: 5.7–18.6%), showing the significant differences across families within taxa. Captivity was associated with a higher prevalence (37.8, 95% CI, 34.3–41.4%) than in wild reptiles (14.8, 95% CI, 11.0–19.6%), highlighting reptiles’ role in the transmission of *Salmonella* to humans (Muslin et al., 2025).

Out of the 33 isolates, 17 *Salmonella* isolates belonged to *Salmonella enterica* sub species *enterica*, and the remaining six isolates belonged to subspecies *diarizonae, arizonae and houten*e (Supplementary Table 1). Out of the *Salmonella* isolates serotyped from captive reptiles, 10 isolates were identified as Salmonella Braenderup, *Salmonella* Sandiego, *Salmonella* Newport, *Salmonella* Oranienburg, *Salmonella* Kisarawe, *Salmonella* Saintpaul (Supplementary Table 1), and the isolate originating from a wild reptile was *Salmonella* Gaminara (Table 1). The remaining 13 *Salmonella* isolates from captive reptiles were not assigned to a known serovar, indicating the diversity of non-typhoidal *Salmonella* in captive reptiles (Supplementary Table 1).

### 4.2 Phenotypic and genotypic AMR analysis

Among the studied *Salmonella* isolates, 62.5% were MDR. Mainly, the resistance was observed against Aminoglycosides and beta-lactams (Figure 1; Table 2). Genomic analysis confirms the phenotypic resistance, but most of the AMR genes observed were against fluoroquinolones, carbapenems, quinolones, etc. (Figure 3; Supplementary Table 2). Resistance genes may be present but not expressed due to various regulatory factors, including epigenetic mechanisms, repressor or activator proteins controlling gene transcription, phenotypic adaptation, mutations, and environmental conditions such as pH, temperature, and nutrients that affect gene activation (Davies and Davies, 2010; Wright, 2007). However, these findings indicate a potential risk for developing phenotypic resistance by *Salmonella* in reptiles, as fluoroquinolones and third- generation cephalosporins used to be the most used antimicrobials in reptiles (Isaza and Jacobson, 2013; Hernandez-Divers et al., 2017).

The identification of *repA* gene in three *Salmonella enterica* WGS datasets provides evidence of the presence of plasmid-derived sequences, as *repA* gene is the key protein involved in the initiation of plasmid DNA replication and stable inheritance (Del Solar et al., 1998; Thomas and Nielsen, 2005). Furthermore, *repA* is a conserved gene across plasmid replicons and is commonly used as a marker for plasmid identification. However, detection of *repA* alone is insufficient to assign a specific plasmid incompatibility (Inc) group, as similar replication genes are widely distributed among diverse plasmid families (del Solar et al., 1998; Thomas and Nielsen, 2005). This finding further insists that the additional plasmid replicon typing or plasmid reconstruction methods are required for an accurate plasmid classification based on the WGS data, where fragmented assemblies and plasmid diversity could limit precise identification (Robertson et al., 2023).

### 4.3 Plasmid DNA analysis

The detection of the protein family related to *tetA* gene in the WGS analysis and *tet*A gene on the plasmid of tetracycline and doxycycline resistance SAL-21-VL-NY-FL-0013 isolated from a pet python that died from a GIT infection is of particular concern because it represents a highly mobile tetracycline resistance determinant capable of rapid horizontal dissemination. This highlights the role of pet reptiles as stable zoonotic reservoirs of highly mobile genetic elements (Figure 2; Table 3). Further, the *tetA*-encoded efflux pump actively expels tetracycline from the bacterial cell, conferring clinically relevant resistance, and its plasmid localization facilitates transfer across strains, serovars, and even bacterial species (Chopra and Roberts, 2001; Roberts, 2005).

Studied isolates of plasmid DNA showed stability, yet mobilizable plasmids may serve as reservoirs for virulence and adaptation traits. Stability systems support the long-term maintenance of virulence and resistance plasmids in asymptomatic reptiles, whereas phage-related recombination and site-specific integration promote genetic plasticity and the acquisition of traits associated with invasive disease (Frost et al., 2005; Touchon et al., 2017). The detection of conjugative or mobilizable transfer regions further indicates the potential for horizontal dissemination of these plasmids across bacterial hosts, increasing the risk of transmission of virulence and AMR determinants at the human–animal interface (Partridge et al., 2018).

### 4.4 Transcriptomic AMR analysis

Hundreds of differentially expressed genes are identified in four *Salmonella enterica* isolates grown at the sub-MICs of FAZ and GEN when compared with those grown without antibiotics (Figure 5; Tables 4, 5). Most of the AMR genes identified in the RNA analysis corresponded with resistance determinants identified through WGS analysis (Tables 4, 5). This concordance between genomic and transcriptomic data is consistent with literature demonstrating that WGS reliably predicts many AMR determinants but may not fully explain phenotypic resistance because gene expression levels, regulatory pathways, and environmental stimuli influence the functional expression of encoded genes (Cooper et al., 2020). The identification of novel AMR-associated genes through transcriptomic analysis (Tables 4, 5) highlights the advantage of RNA-seq in revealing inducible or conditionally expressed resistance mechanisms that may not be evident from genomic data alone (Hashemi et al., 2020). Many of the differentially expressed AMR genes identified in this study were classified by the InterPro protein database into protein families associated with MDR, efflux pumps, and membrane transport systems. InterPro integrates predictive models from multiple protein signature databases and is widely used for functional annotation of newly detected proteins and domain families (Paysan-Lafosse et al., 2023). Genes associated with efflux systems were identified and found to be differentially expressed in both antimicrobial-resistant and susceptible isolates, supporting the role of efflux-mediated resistance as a major intrinsic mechanism in Gram-negative bacteria, including *Salmonella* (Yamasaki et al., 2023).

This study revealed that Major Facilitator Superfamily (MFS) proteins were frequently differentially expressed in the isolates (Tables 4, 5). MFS transporters are among the largest families of secondary transporters in bacteria and are widely implicated in MDR by exporting structurally diverse antibiotics out of the cell (Lekshmi et al., 2024). SAL-20-VL-NY-FL-0013 and SAL-25 W-FL-0001 exhibited upregulation of MFS domain-containing proteins when grown with FAZ, suggesting that enhanced efflux activity may reduce intracellular antibiotic accumulation, thereby increasing resistance toward FAZ. Similar efflux-mediated resistance mechanisms have been widely documented in *Salmonella enterica* and other Enterobacteriaceae (Yamasaki et al., 2023; Du et al., 2018). Under FAZ exposure, SAL-21-VL-NY-FL-0013 showed concurrent upregulation of MFS transporter and aminoglycoside N (6′)-acetyltransferase type 1, alongside the downregulation of several other transport-related genes (Table 4). Acetyltransferases modify aminoglycosides by acetylation, thereby reducing antibiotic binding to bacterial ribosomes and conferring resistance to drugs such as gentamicin and amikacin (Magnet et al., 2001). This simultaneous upregulation of efflux and enzymatic resistance mechanisms in this isolate likely underlies its broader resistance phenotype. Conversely, some isolates exhibited downregulation of genes associated with multidrug transport systems, including ABC-type multidrug transporters and the p-hydroxybenzoic acid efflux pump subunit AaeA (Table 4). ABC transporters are ATP-driven membrane proteins that play important roles in drug efflux and cellular detoxification in many bacterial species (Bogomolnaya and Vazquez-Torres, 2013). However, not all transporters function directly as antibiotic exporters, and some primarily transport metabolic substrates and may be differentially regulated during antibiotic stress as part of broader metabolic adaptation. The AaeAB efflux system is primarily associated with the export of aromatic carboxylic acids rather than classical antibiotic resistance (Van Dyk et al., 2004). Nevertheless, transport systems originally involved in metabolic detoxification can contribute indirectly to antimicrobial tolerance or stress adaptation under antibiotic pressure (Van Dyk et al., 2004). Therefore, the differential expression of AaeA observed in several isolates used in this study may reflect shifts in metabolic or membrane-associated stress responses rather than a direct resistance mechanism.

Exposure to GEN induced distinct transcriptional shifts, particularly in the regulation of MFS transporters, membrane proteins, and regulatory elements. In isolate SAL-20-VL-NY-FL-0013, GEN exposure resulted in downregulation of an MFS transporter but upregulation of several other membrane-associated genes, including Protein RarD and AaeA. This indicates that *Salmonella* selectively activates different transporter systems based on the specific antibiotic stress encountered. Such compensatory regulation among efflux systems has been reported in multidrug-resistant bacteria, where multiple transporters collectively contribute to antibiotic tolerance (Yamasaki et al., 2023). Notably, the GEN-susceptible isolate SAL-24-VL-NY-FL-0008 exhibited widespread downregulation of resistance-related genes, including MFS transporters and aminoglycoside acetyltransferase, suggesting a lack of an effective transcriptional response to aminoglycoside exposure. The reduced expression of aminoglycoside-modifying enzymes or efflux systems has previously been associated with increased susceptibility to aminoglycosides in Gram-negative bacteria (Magnet et al., 2001). The differential expression of TetR-family transcriptional regulators observed in *Salmonella* in this study may also modulate antibiotic resistance. TetR-family regulators control numerous genes involved in efflux pumps, metabolic pathways, and stress responses in Gram-negative bacteria (Cuthbertson and Nodwell, 2013). Changes in TetR expression may therefore influence the expression of downstream resistance determinants, contributing to the transcriptional response observed in the studied isolates.

The findings of this study demonstrate that exposure to FAZ and GEN induces transcriptional responses in *Salmonella* involving efflux systems, transport proteins, regulatory genes, and enzymatic resistance determinants. The combination of WGS and transcriptomic analyses provides complementary insights into AMR by identifying both the genetic potential for resistance and the genes actively expressed during antibiotic stress. Such integrative approaches are essential for understanding the complex regulatory networks underlying AMR in bacterial pathogens (Cooper et al., 2020).

### 4.5 Virulency and public health importance of *Salmonella enterica* isolates associated with reptiles

This study revealed the presence of genes related to fimbrial adhesion in *Salmonella enterica* isolates, namely fimbrial assembly (*faeC*), Fimbrial usher protein (*faeD*), and fimbrial chaperone (*faeE*), in two of the captive reptiles that had GIT infection, and one of the captive reptiles had systemic infection, but none in the healthy reptiles. Even though there is very limited information specifying the well-described role of *faeC*, *faeD*, and *faeE* as virulence in *Salmonella* in the scientific literature, the function of these genes is well characterized in enterotoxigenic *Escherichia coli* (ETEC) and related bacteria, as these genes are critical in fimbrial adhesion, colonization to cause diarrheal disease in animals (Crofts et al., 2018; Van den Broeck et al., 2000). The presence of these three genes in *Salmonella* isolates involved in clinical infections indicates that they were acquired by *Salmonella* from other gut pathogens via horizontal gene transfer (Bolotin and Hershberg, 2017). One of the isolates, SAL-24-VL-NY-FL-0008, which showed protein families related to *faeC*, *faeD*, and *faeE* in WGS analysis, was included in the transcriptomic study. However, genes expressed in both the SAL-24-VL-NY-FL-0008 treatment and control groups did not include those induced by host-specific environmental cues, rather than standard *in vitro* culture conditions (Nikaido et al., 2012; Result not presented).

The current study also observed the presence of the *manB* gene only in a *Salmonella enterica* isolate from a reptile that died of a systemic infection (Supplementary Table 3). *Man*B gene encodes an enzyme called phosphomannomutase (PMM) that critical for synthesizing mannose- containing surface structures in *Salmonella*, including the surface lipopolysaccharide (LPS) O- antigen which is essential for host immune evasion, resistance to complement, phagocytosis and production of colonic acid, an extracellular polysaccharides that can shield the cell surface and affect response (Jensen and Reeves, 2001). The *manB* gene itself is not a direct “virulence gene” like SPI effectors, but it supports systemic infection by enabling the synthesis of key surface polysaccharides such as LPS O-antigen (Li et al., 2017). These structures protect *Salmonella* from bloodstream survival and immune evasion, which remain critical to systemic infection success (Li et al., 2017).

Additionally, we report the detection of virulence *tvi* and *vex* genes, which constitute the *viaB* locus responsible for Vi capsular antigen biosynthesis and export, in a single *Salmonella enterica* isolate (SAL-20-VL-NY-FL-0001) originating from a pet python that died of a systemic infection (Supplementary Table 3). This finding is unexpected, as the Vi antigen has classically been regarded as a defining feature of human-adapted, typhoidal *Salmonella* such as *S. typhi* and *S. paratyphi C* and is largely absent from reptile-associated non-typhoidal *Salmonella* (AS) lineages (Boyd et al., 1998; Raffatellu et al., 2006). Reptiles are well-established reservoirs of diverse non-typhoidal *Salmonella* serovars, which typically colonize the intestinal tract asymptomatically and only occasionally cause systemic disease in their ectothermic hosts (Mitchell and Shane, 2001; Mermin et al., 2004). Genomically, these reptile-associated strains generally lack *Salmonella* Pathogenicity Island 7 (SPI-7), which carries the *viaB* locus encoding Vi antigen synthesis (*tvi*) and export (*vex*) genes (Boyd et al., 1998). Therefore, the identification of *tvi* and *vex* genes in Reptile-Associated *Salmonella* (RAS) isolates represents a marked deviation from the canonical RAS genomic structure. The Vi capsule is a well-characterized virulence determinant that confers immune-evasion properties, comprising resistance to complement-mediated killing, reduced opsonophagocytosis, and suppression of innate inflammatory signaling (Wilson et al., 2008; Raffatellu et al., 2006). In *S. typhi*, Vi antigen expression facilitates survival in the bloodstream and systemic dissemination while limiting intestinal inflammation, a hallmark of typhoid fever (Guerra et al., 2025). The presence of Vi-associated genes in reptile isolates may therefore explain the systemic infection observed in the affected python, suggesting the acquisition of virulence traits typically associated with invasive disease. Future studies should focus on basic functional analysis of the *tvi* and *vex* genes to validate the presence of such human-adapted, typhoidal *Salmonella* gene elements and to assess the associated zoonotic risks.

From an evolutionary perspective, these outcomes raise the possibility of horizontal gene transfer (HGT) of the viaB locus or SPI-7 between *Salmonella* lineages. SPI-7 is a large, mosaic pathogenicity island, and previous studies have suggested that it was acquired through horizontal transfer during the evolution of typhoidal *Salmonella* (Boyd et al., 1998; Pickard et al., 2003). Acquisition of this locus by reptile-associated strains would demonstrate ongoing exchange of virulence determinants across ecological and host boundaries, with potential consequences for host-range expansion and virulence. Vi-positive *Salmonella* strains are associated with increased invasiveness and systemic disease in humans, and their emergence in exotic pet reservoirs could elevate the risk of severe or atypical zoonotic infections. Moreover, the presence of Vi antigen may complicate routine diagnostic interpretation, as Vi expression is often used as a marker of typhoidal *Salmonella* (Raffatellu et al., 2006).

Simultaneously, several limitations should be acknowledged with this finding. Genomic detection of the *tvi* and *vex* genes alone does not confirm functional expression of the Vi capsule in the isolate. Environmental aspects, including temperature and host niche, may influence capsule expression, particularly in ectothermic hosts. Future studies should include phenotypic confirmation of Vi expression, transcriptional analyses, and long-read sequencing to confirm the integrity, genomic context, and mobility of the *viaB* locus. Comparative phylogenomic analyses will be essential to determine whether these isolates represent sporadic acquisition events or an emerging invasive lineage.

In conclusion, the detection of mobile resistance determinants, including genomic and plasmid-associated antimicrobial genes, together with multiple efflux and regulatory systems, highlights the potential for horizontal and vertical dissemination of AMR. Furthermore, the presence of numerous virulence-associated genes involved in adhesion, invasion, immune evasion, and secretion systems indicates that reptile-associated *Salmonella* plausibly may have the capacity to cause severe diseases in humans. The results of this study indicate that reptiles serve as reservoirs of multidrug-resistant and potentially virulent *Salmonella,* signifying the importance of continued surveillance and strengthened hygiene measures in handling reptiles to reduce the risk of RAS in people.

## Data Availability

The data presented in the study are deposited in the National Center for Biotechnology Information (NCBI) repository under BioProject numbers PRJNA503850, PRJNA1331838, and PRJNA1425458.

## Footnotes

1
https://github.com/rrwick/poechop
